# Supplementary material for: A universal and independent synthetic DNA ladder for the quantitative measurement of genomic features
Source: Nat Commun. 2020 Jul 17;11:3609. doi: 10.1038/s41467-020-17445-5 (PMC7367866; doi:10.1038/s41467-020-17445-5)
Supplement: Supplementary file 2 — Supplementary Information [file 41467_2020_17445_MOESM2_ESM.pdf]

A universal and independent synthetic DNA ladder for the quantitative measurement of genomic features.

Reis et al.

Supplementary Figures

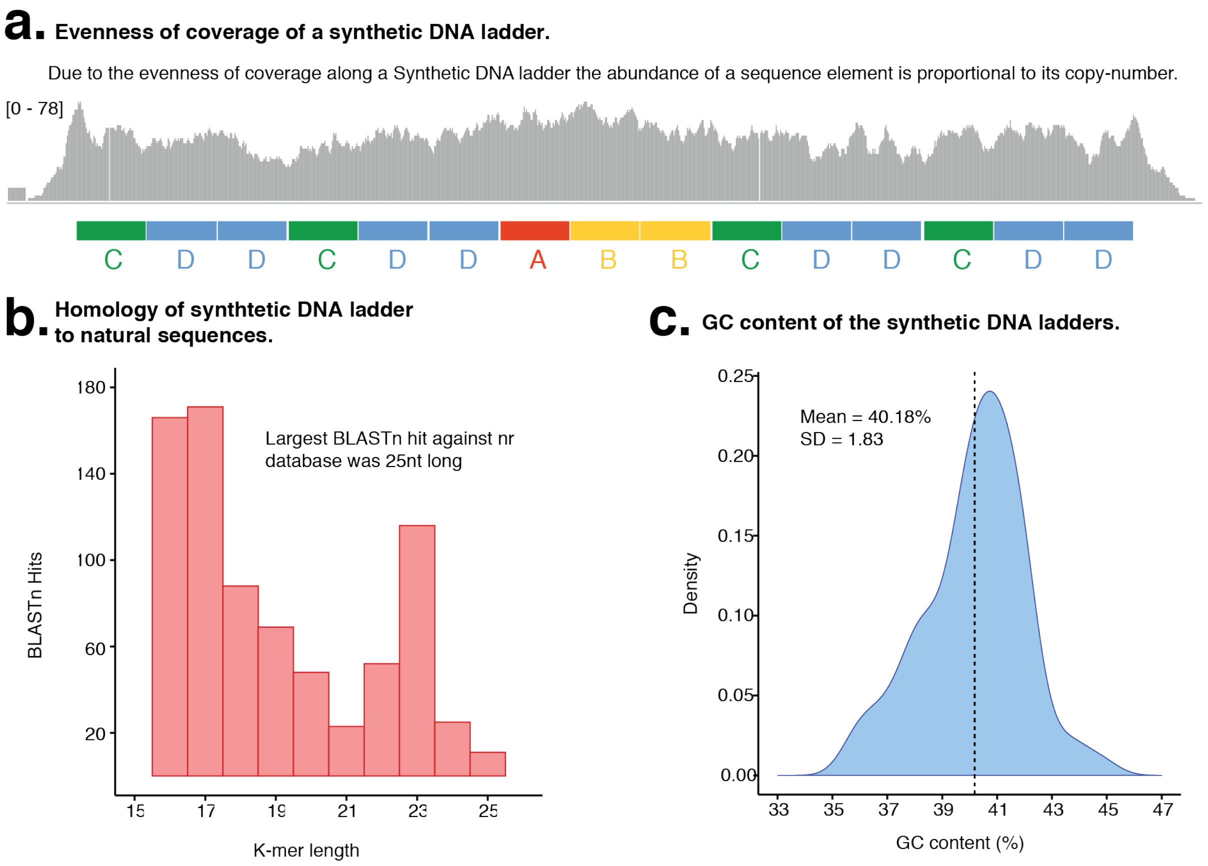

Supplementary Figure 1. (**a**) Histogram showing the read depth along a Synthetic DNA ladder with the corresponding sequence elements indicated below. (**b**) Size distribution of BLASTn hits for a search of all n=56 independent DNA ladder sequence elements against the non-redundant nucleotide database with a word size of 16 nt. The largest observed hit was 25 nt long. (**c**) GC content (%) distribution across all n=56 DNA ladder sequence elements. The dashed line represents the average GC content (%) observed in the DNA ladders.

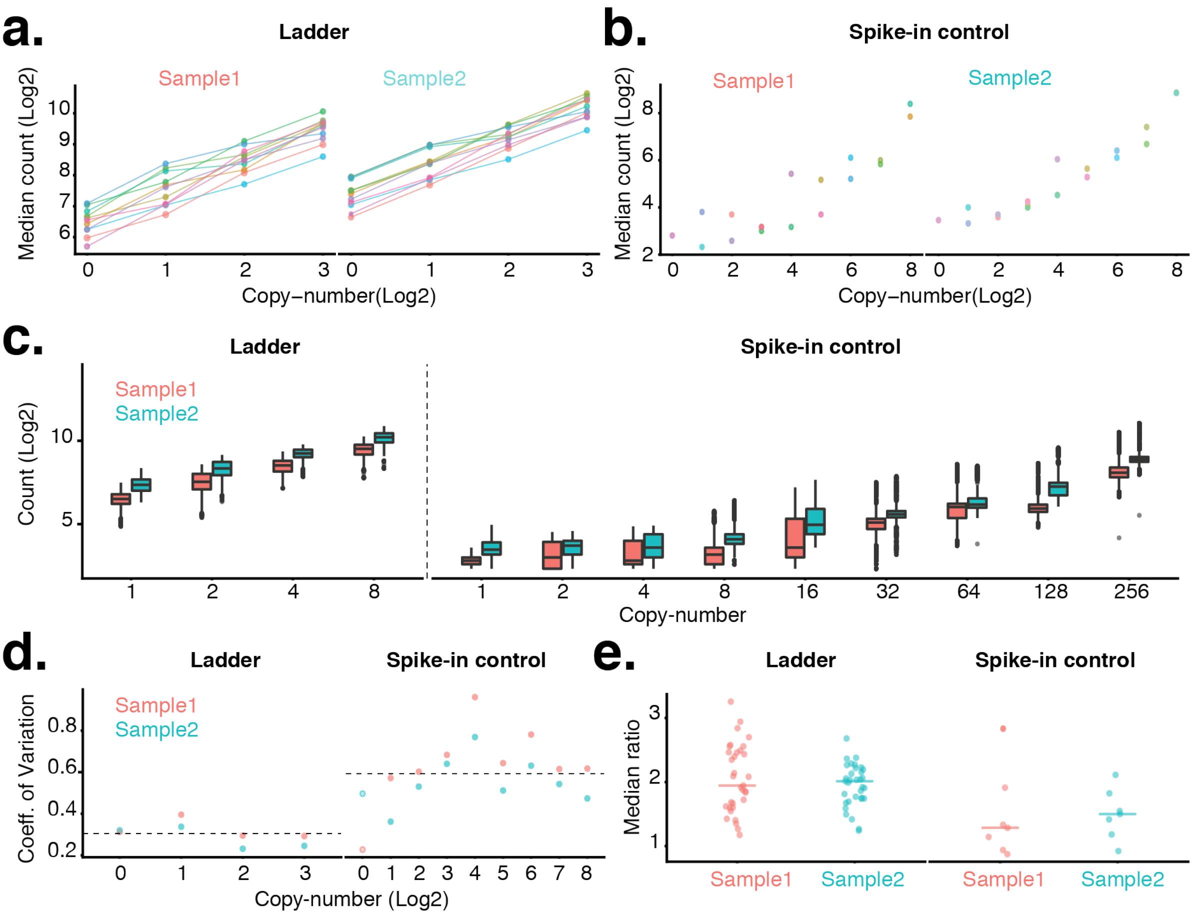

Supplementary Figure 2. (**a**) Scatter plots show the observed abundance (in median k-mer counts) of sequence elements versus expected copy-number in the synthetic DNA ladder. Two independent libraries are shown (Sample1=orange and Sample2=green); (**b**) Scatter plots show the observed abundance (in median k-mer counts) of individual spike-ins versus expected copy-number; (**c**) Box-whisker plots show the k-mer count distribution for two libraries (containing the synthetic DNA ladders, spike-in controls) that were independently prepared and sequenced to differing depths; (**d**) Scatter plot shows the coefficient of variation at different copy-numbers for the ladder and other spike-ins (Hardwick et al., 2018) when considering all k-mer counts in the two independent libraries, with the dashed lines indicating the corresponding means. (**e**) The plot shows the median ratios between subsequent copy-numbers for individual synthetic DNA ladders and spike-ins in the two independent libraries, with horizontal line indicating the mean. In **c**, where data is represented as boxplots, the middle line is the median, the lower and upper hinges correspond to the first and third quartiles, the upper whisker extends from the hinge to the largest value no further than 1.5 x IQR from the hinge (where IQR is the inter-quartile rage) and the lower whisker extends from the hinge to the smallest value at most 1.5 x IQR of the hinge and any points beyond the whiskers are represented individually. In **a-e**, the statistics were calculated for n=14 independent synthetic ladders and n=17 spike-in controls (Hardwick et al., 2018), over 2 independent experiments.

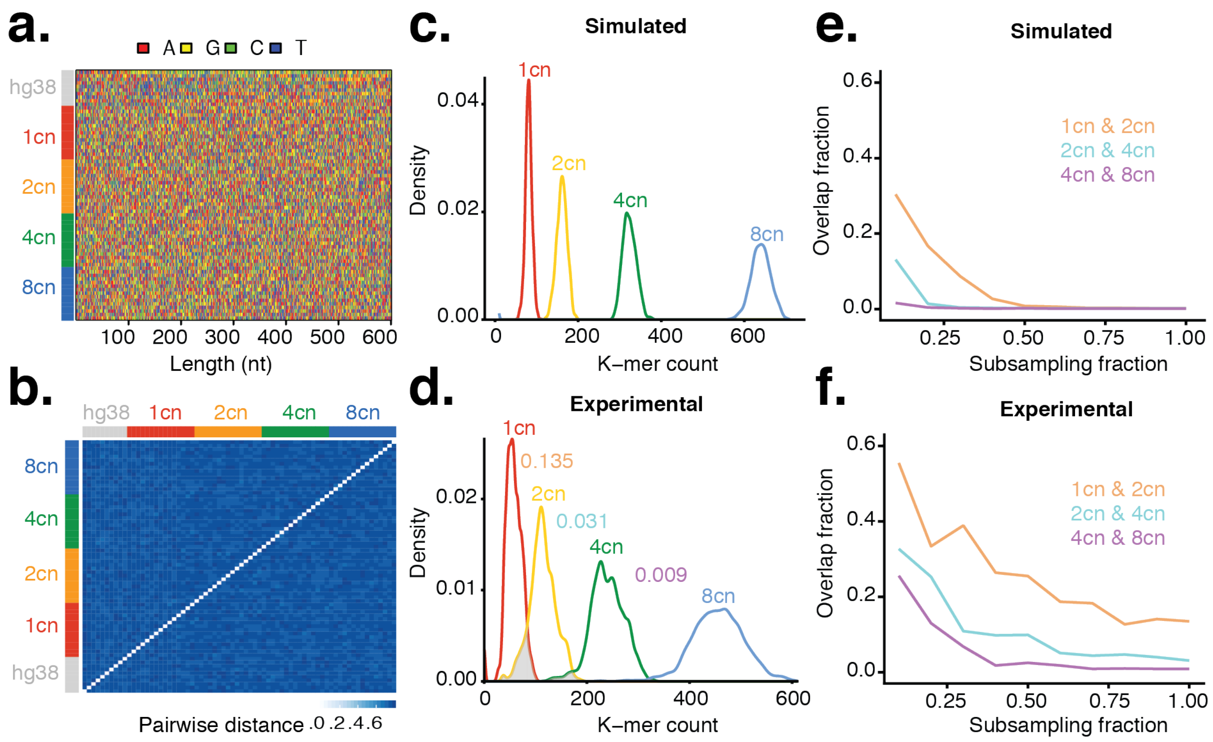

Supplementary Figure 3. (**a**) Matrix comparison of nucleotide sequence between all sub-sequence elements in the synthetic ladders (1, 2, 4 and 8cn) and also ten human sequences of equal length (600nt). Each nucleotide is encoded with a different color (A=red, G=yellow, C=green and T=blue); (**b**) Pairwise matrix comparison showing the distance between sub-sequence elements in the synthetic ladders and hg38 sequences, where distance can range from 0 (identical) to 1 (completely dissimilar). (**c** and **d**) Density distribution of k-mer counts for each cn unit (1cn=red, 2cn=yellow, 4cn=green and 8cn=blue) in the ladder in a simulated and experimental (HiSeq X Ten/PCR-free) libraries, respectively. The fraction overlapping between successive copy-number units is indicated in gray. (**e** and **f**) The fraction of k-mers overlapping between subsequent copy-numbers decreases relative to increasing variation due to decreasing library depth (by subsampling). In **c** and **d**, the densities were calculated for n=14 independent synthetic ladders.

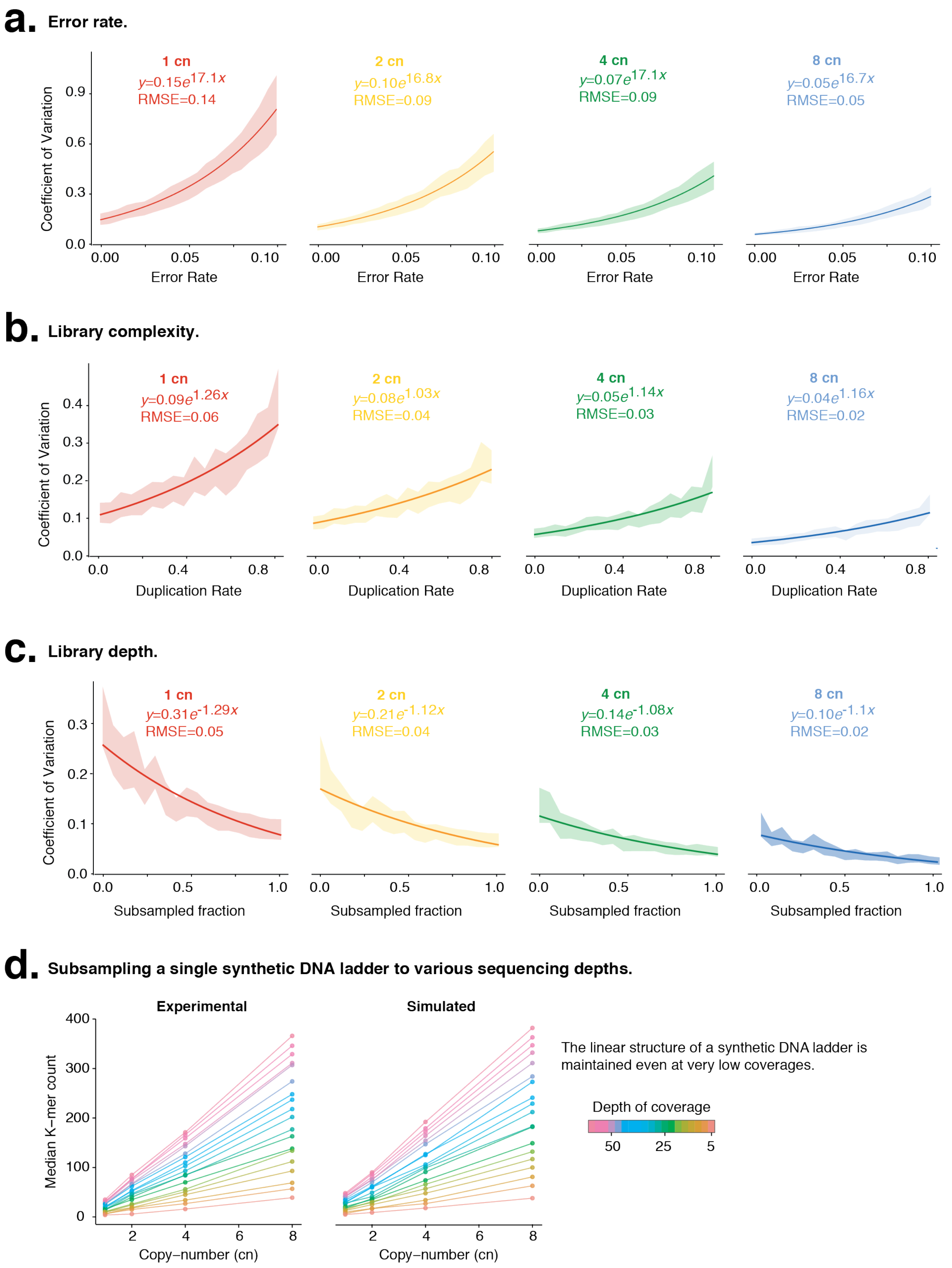

Supplementary Figure 4. Modeling the impact of common NGS technical variables such as error rate (**a**), library complexity (**b**) and library depth (**c**) on the variability observed in subsequent cn units as measured by the coefficient of variation. For each unit, the line of best fit is shown with the underlying formula and the bands around the line represent the error, with the corresponding RMSE also indicated. (**d**) Plot showing copy-number (cn) versus median k-mer count for a single synthetic DNA ladder subsampled from 55x coverage down to 5x in an experimental and simulated libraries. In **a-c**, the coefficient of variation was calculated across all n=14 independent synthetic DNA ladders.

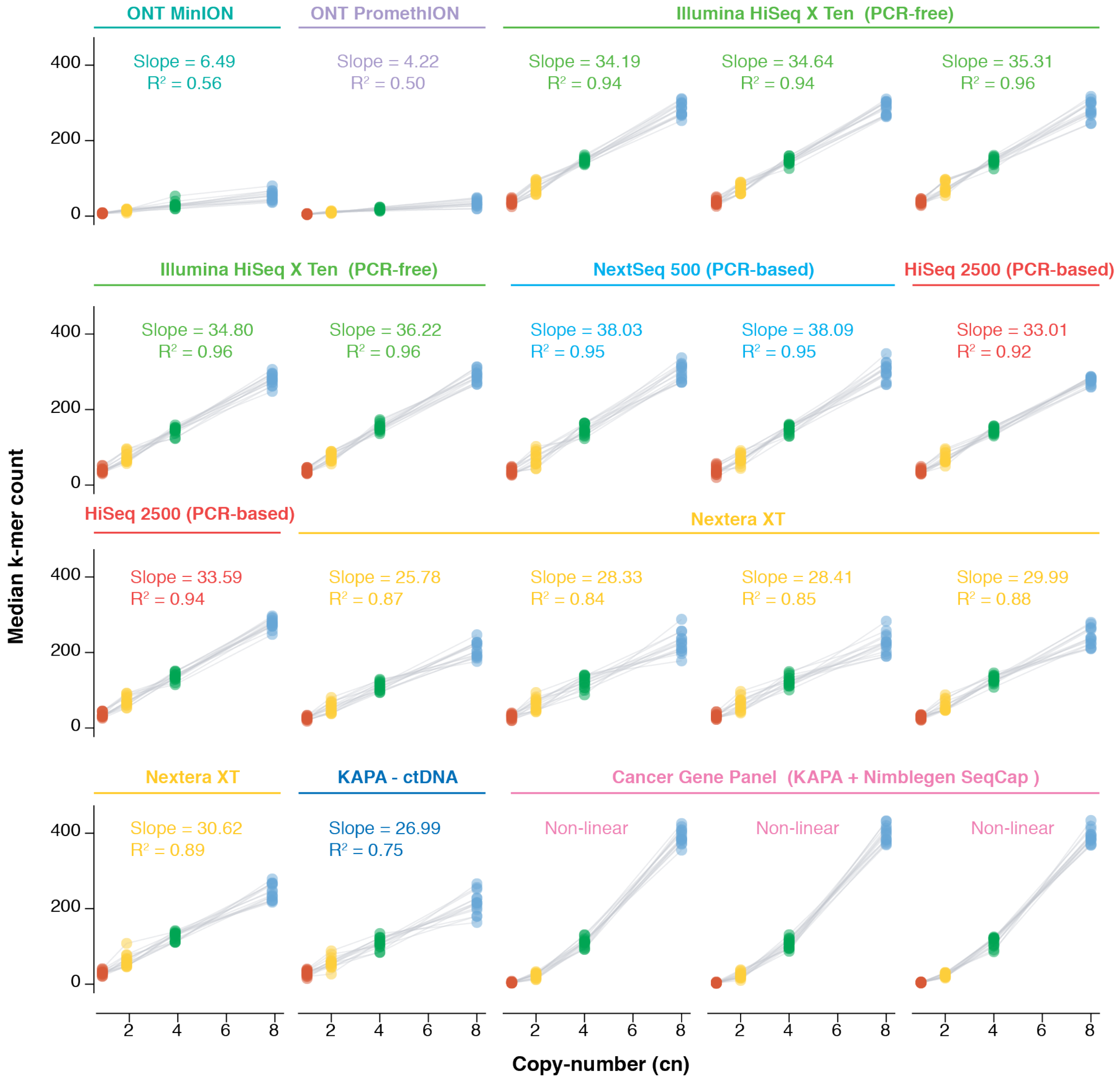

Supplementary Figure 5. The panel shows the median k-mer count versus cn unit for the DNA ladders in multiple samples generated with different sequencing technologies (e.g. ONT Nanopore MinION, ONT Nanopore PromethION, Illumina HiSeq X Ten, Illumina NextSeq 500 and Illumina HiSeq 2500 intruments) or prepared with alternative protocols (e.g. KAPA HyperPlus PCR-free, KAPA HyperPlus PCR-based, KAPA and Nextera XT kits, or target enrichment by oligonucleotide hybridisation). The regression slope and R^2 are indicated for all samples with the exception of targeted capture libraries, in which the DNA ladder does not have a linear structure. In each library the statistics were calculated for n=14 independent synthetic ladders.

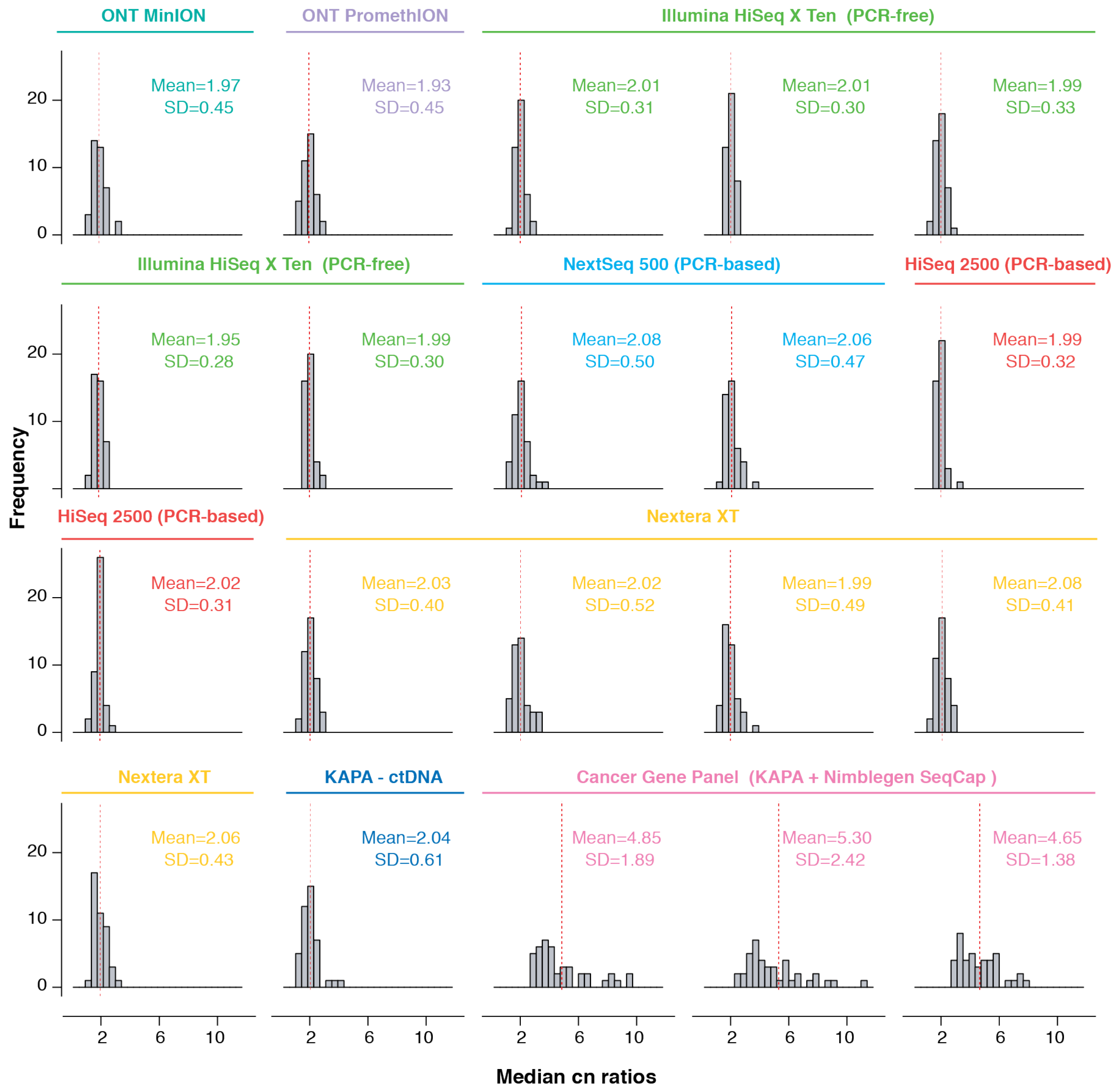

Supplementary Figure 6. The panel shows the distribution of median cn ratios for the DNA ladders in multiple samples generated with different sequencing technologies (e.g. ONT Nanopore MinION, ONT Nanopore PromethION, Illumina HiSeq X Ten, Illumina NextSeq 500 and Illumina HiSeq 2500 instruments) or prepared with alternative protocols (e.g. KAPA HyperPlus PCR-free, KAPA HyperPlus PCR-based, KAPA and Nextera XT kits, or target enrichment by oligonucleotide hybridisation). With the exception of targeted capture libraries, in which the DNA ladder does not have a linear structure, the distributions are approximately centered around 2. In each library the statistics were calculated for n=14 independent synthetic ladders.

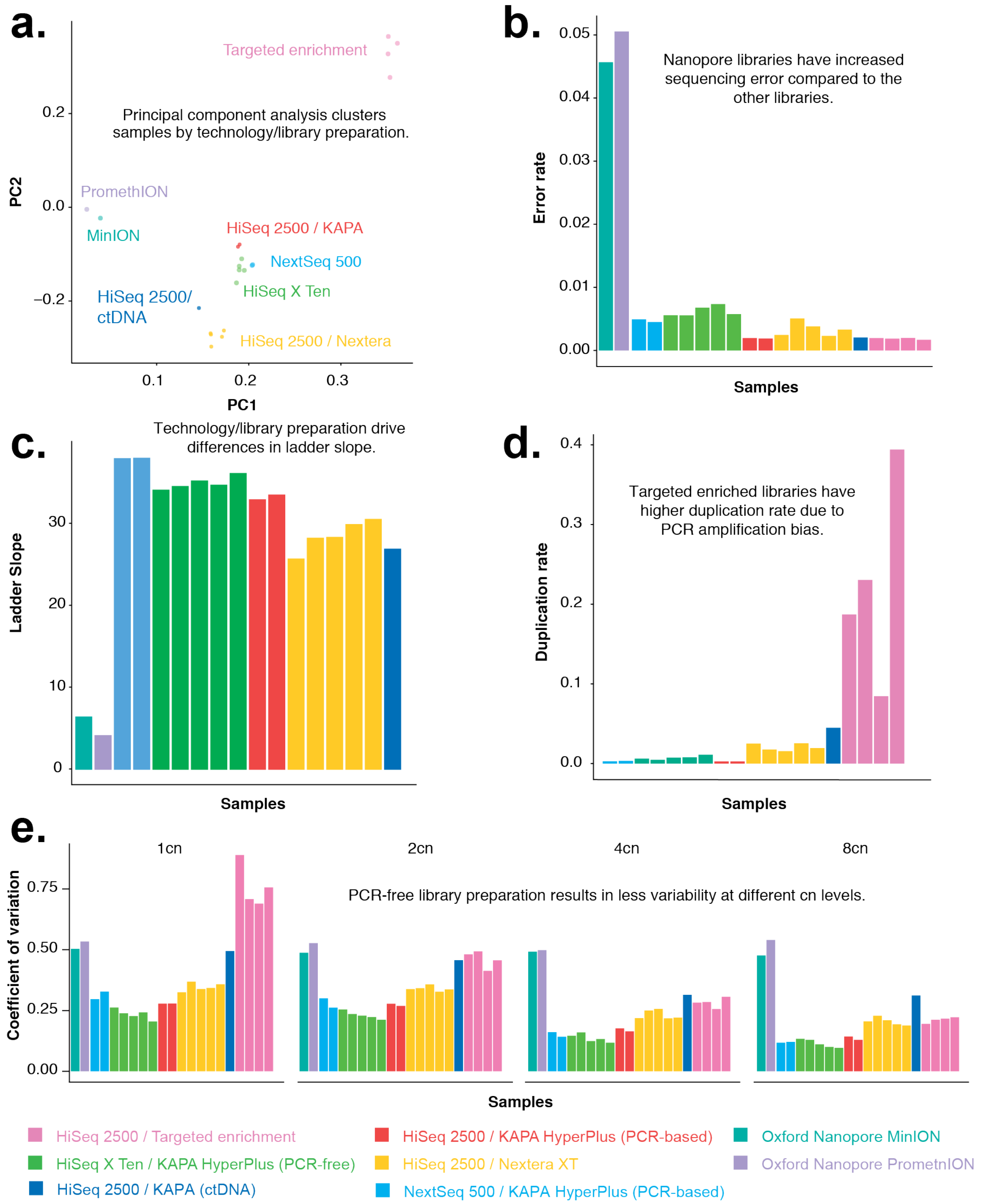

Supplementary Figure 7. (**a**) PCA plot of DNA ladder k-mer counts from experimental libraries prepared with different kits (e.g. KAPA HyperPlus PCR-free, KAPA HyperPlus PCR-based, KAPA and Nextera XT kits, or target enrichment by oligonucleotide hybridisation) and sequenced with different technologies (e.g. ONT Nanopore MinION, ONT Nanopore PromethION, Illumina HiSeq X Ten, Illumina NextSeq 500 and Illumina HiSeq 2500 instruments). Barplots showing the sequencing error rate (**b**), DNA ladder regression slope (**c**), duplication rate (**d**) and coefficient of variation (**e**) at different cn units for the different experimental libraries. The statistics in **b-e** were calculated across all n=14 independent synthetic DNA ladders.

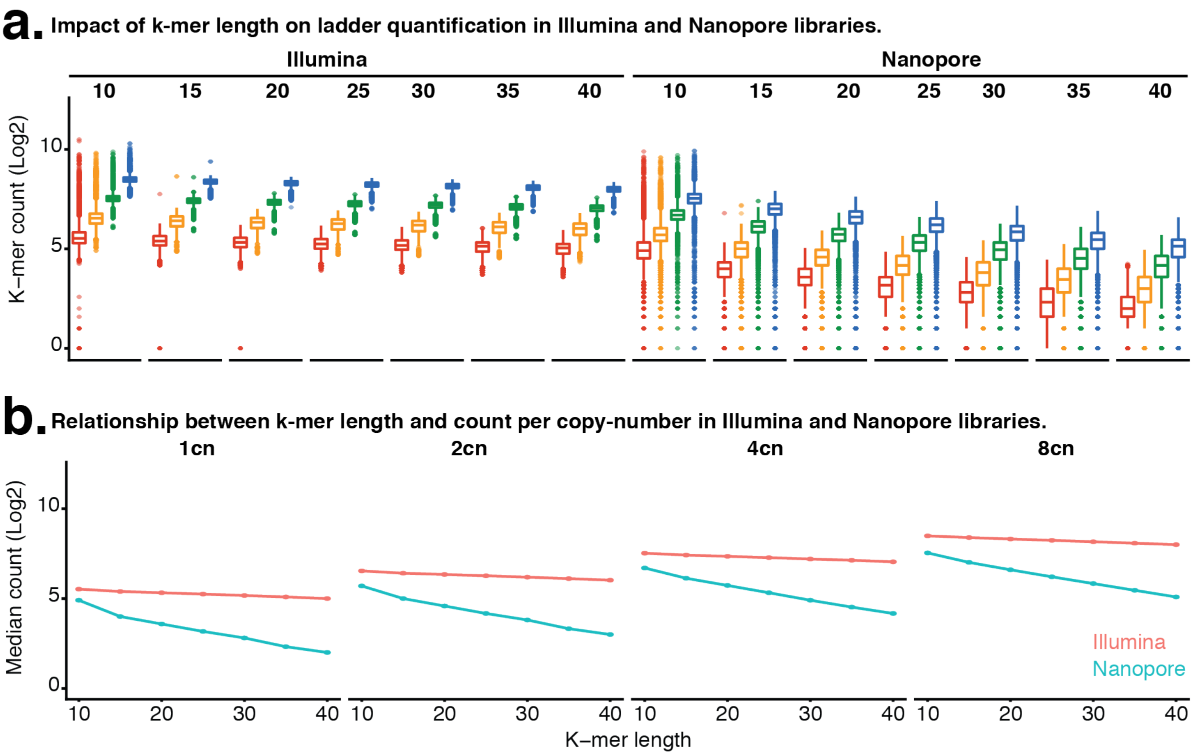

Supplementary figure 8. (**a**) Boxplots show the k-mer count distribution for the different cn units in the ladder (1cn=red, 2cn=yellow, 4cn=green and 8cn=blue) in Illumina and Nanopore libraries as the size of k increases. (**b**) The plot shows the linear relationship between k-mer length and the median observed count for the different cn units in Illumina and Nanopore libraries. The results in **a** and **b** were calculated across all n=14 independent synthetic ladders. In **a**, where data is represented as boxplots, the middle line is the median, the lower and upper hinges correspond to the first and third quartiles, the upper whisker extends from the hinge to the largest value no further than 1.5 x IQR from the hinge (where IQR is the inter-quartile rage) and the lower whisker extends from the hinge to the smallest value at most 1.5 x IQR of the hinge and any points beyond the whiskers are represented individually.

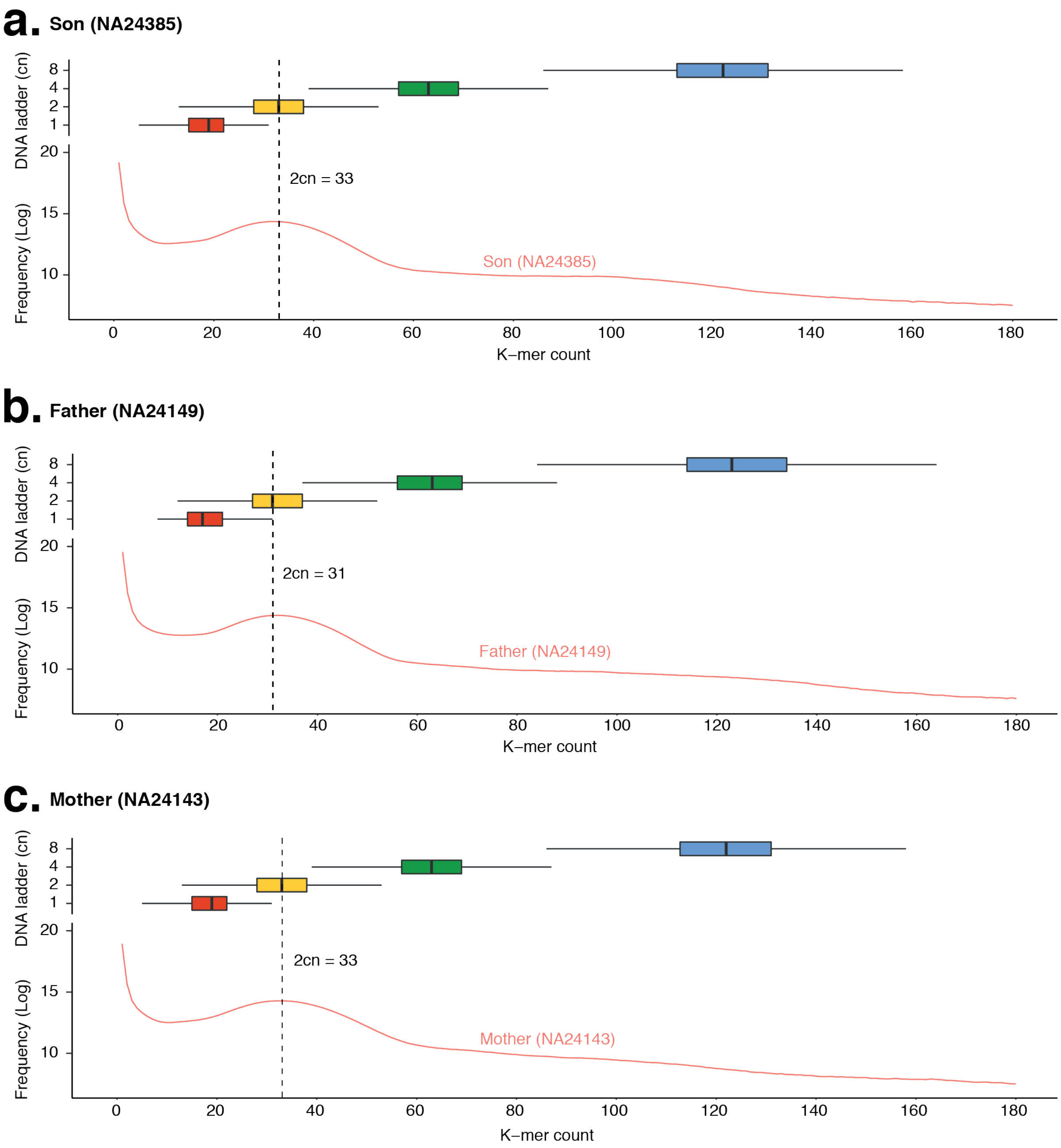

Supplementary Figure 9. Box-whisker plots indicate the k-mer count distribution associated with each cn unit in the DNA ladder (upper panel) in comparison to the k-mer count distribution for the accompanying human genome (bottom panel) in each sample of the Jewish trio: (**a**) Son (NA24385), (**b**) Father (NA24149) and (**c**) Mother (NA24143). The dashed line indicates the median count for k-mers in the human sample, which are calibrated to the median count of 2cn in the accompanying DNA ladder. The boxplots in **a**-**c** were calculated across all n=14 independent synthetic ladders, while the densities represent the k-mer count distribution for k-mers in chr21 in each sample of the Jewish trio. In **a-c**, where data is represented as boxplots, the middle line is the median, the lower and upper hinges correspond to the first and third quartiles, the upper whisker extends from the hinge to the largest value no further than 1.5 x IQR from the hinge (where IQR is the inter-quartile rage) and the lower whisker extends from the hinge to the smallest value at most 1.5 x IQR of the hinge and any points beyond the whiskers are represented individually.

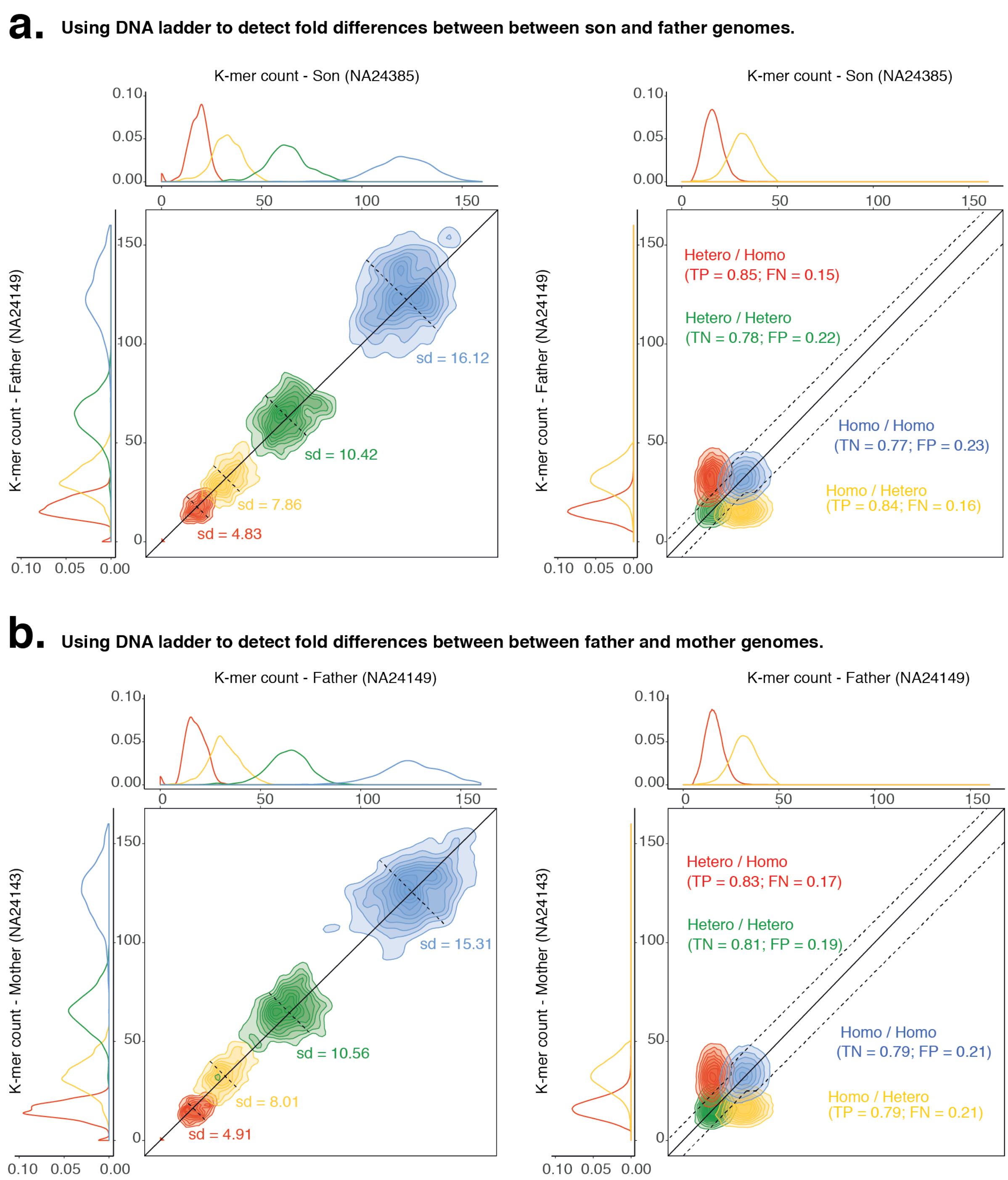

Supplementary Figure 10. (**a**) (**left**) Scatter plot shows ladder k-mer counts in the son (NA2385) and father (NA24149) with the cross-sample variability at each cn level indicated by the standard deviation; (**right**) scatter plot shows germline variants in the son (NA2385) and father (NA24143) genomes grouped by genotype. Dashed lines indicate the limits of significant fold-differences in each genotype group determined based on the variation estimated from the DNA ladders (one-sided t-test; significance = 0.05). The performance is indicated by the fraction of true positives (TP) and false negatives (FN) or true negatives (TN) and false positives (FP). (**b**) Same analysis, but comparing father (NA24143) and mother (NA24143). In **a-b**, the statistical test used was a one-sided t-test and P-values were adjusted for a false discovery rate (FDR) of 0.05.

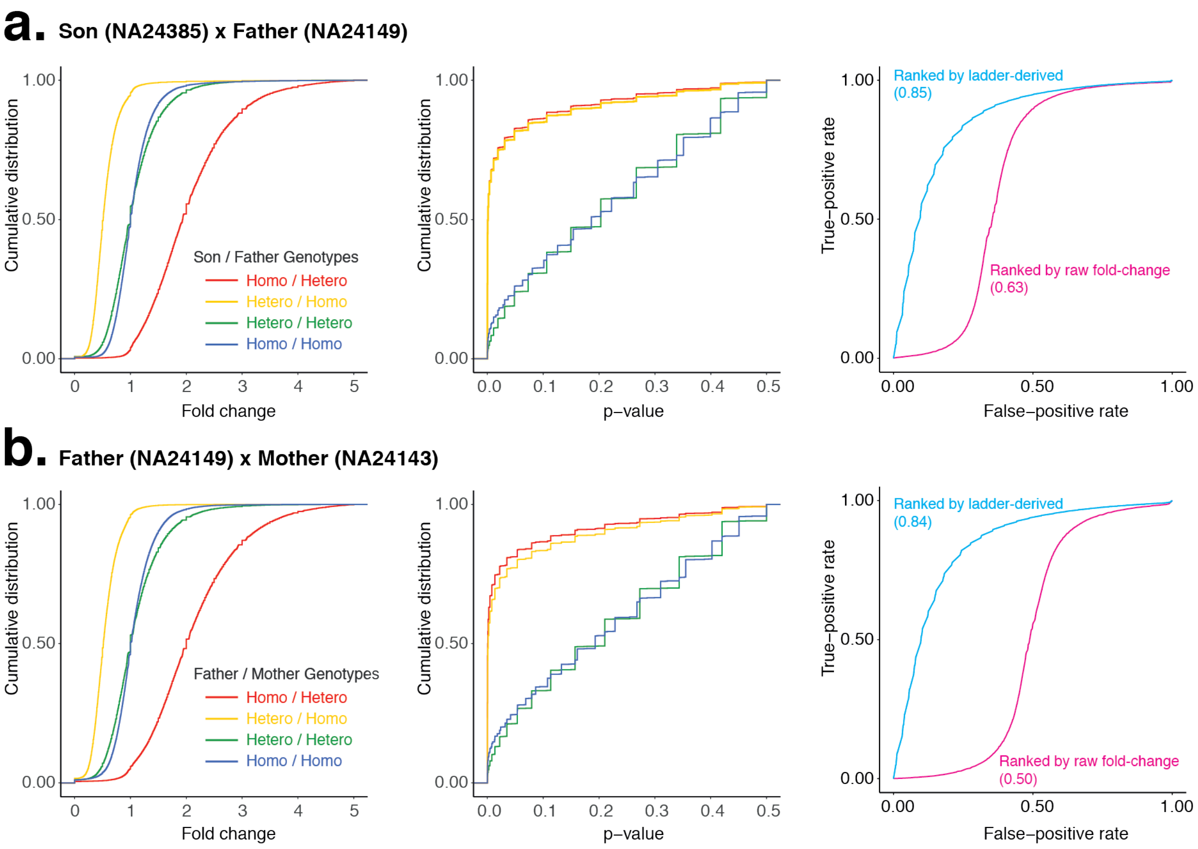

Supplementary Figure 11. (**a**) Cumulative distributions of fold-changes and p-values calculated from the DNA ladder for variant k-mers in the son (NA24385) and father (NA24149) colored by genotype. The ROC curves ranking true-positive and true-negative variant k-mer counts either by fold-change (magenta) or the p-values (one-sided t-test; light blue) estimated from the DNA ladder with the Area Under the Curve (AUC) indicated. (**b**) Same analysis, but comparing father (NA24149) and mother (NA24143). In **a-b**, the statistical test used was a one-sided t-test and P-values were adjusted for a false discovery rate (FDR) of 0.05.

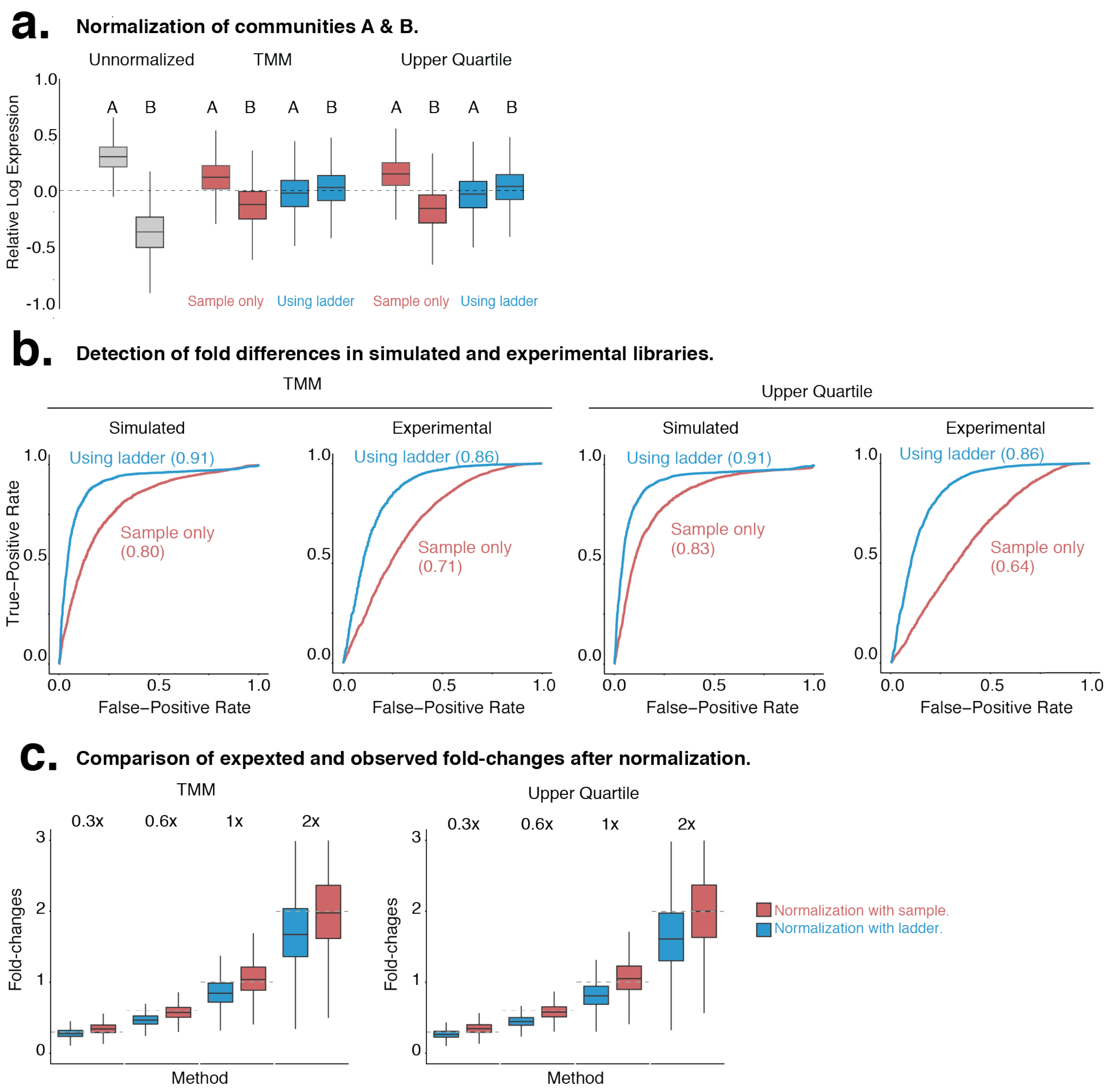

Supplementary Figure 12. (**a**) RLE plots illustrate the impact of normalizing the libraries based on the DNA ladder (blue) or sample (red) k-mer counts using TMM or Upper Quartile methods. (**b**) ROC plots indicate the detection of known fold-change differences between the mock communities A & B following normalization with the synthetic ladder (blue), sample (red), or unnormalized (grey) in simulated or experimental libraries when using TMM or Upper Quartile methods. (c) The boxplots show the observed k-mer count fold-differences between communities A & B following normalization with the DNA ladder (blue) or sample (red) using TMM or Upper Quartile methods, at different expected fold-changes (0.3, 0.6, 1 and 2) represented by horizontal gray lines. A total of 19,768 k-mers from 9 different bacterial species (see Methods) mixed at known fold changes were used to evaluated the normalization performance between communities A & B. A total of 19,768 k-mers from 9 different bacterial species (see Methods) mixed at known fold changes were used to evaluated the normalization performance between communities A & B. In a and c, where data is represented as boxplots, the middle line is the median, the lower and upper hinges correspond to the first and third quartiles, the upper whisker extends from the hinge to the largest value no further than 1.5 x IQR from the hinge (where IQR is the inter-quartile rage) and the lower whisker extends from the hinge to the smallest value at most 1.5 x IQR of the hinge and any points beyond the whiskers are represented individually.

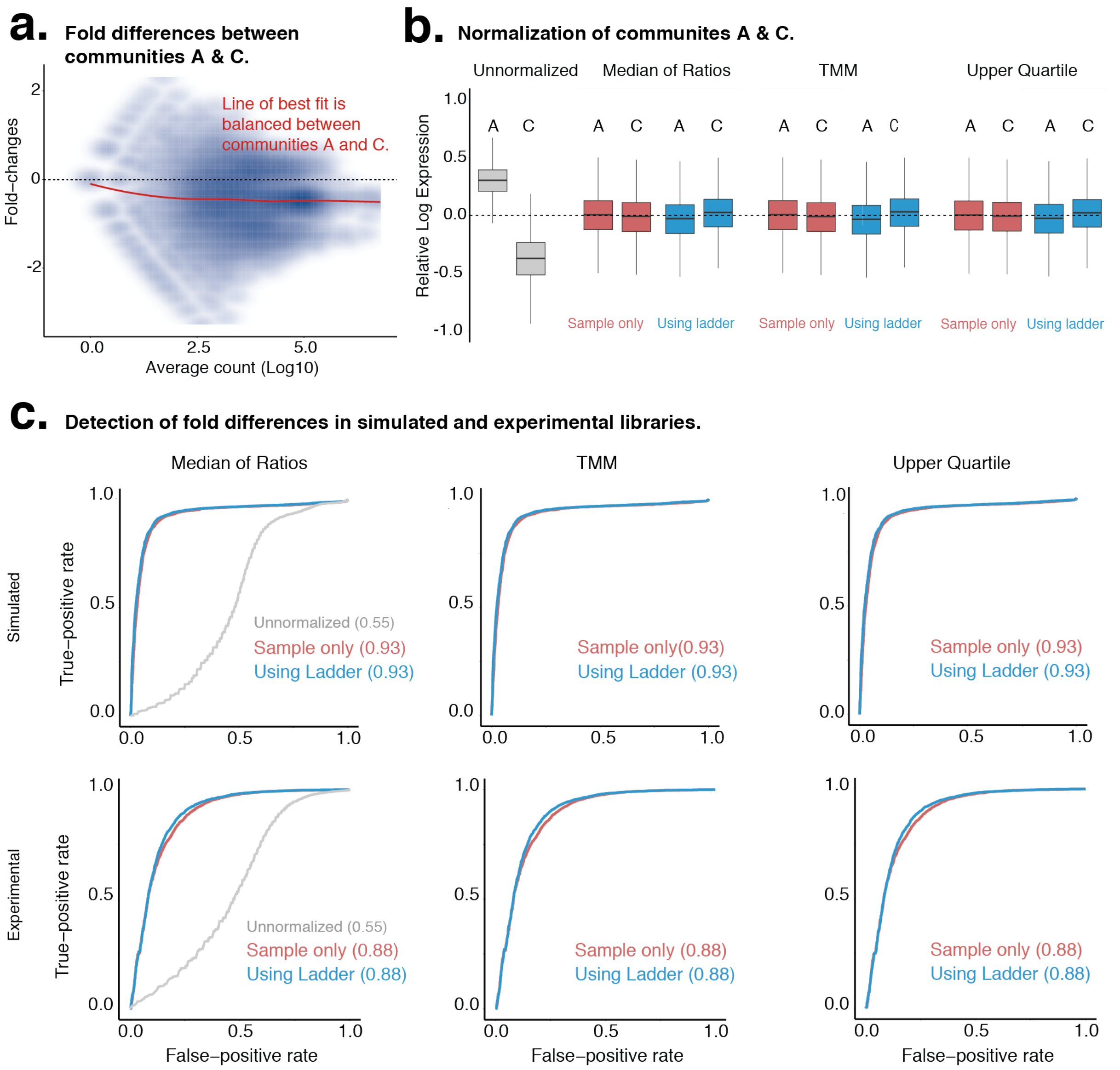

Supplementary Figure 13. (**a**) Scatter-plot of log fold-changes between communities A and C with the line of best fit indicating balanced differences between them. (**b**) RLE plots illustrate the impact of normalizing the samples based on the DNA ladder (blue) or sample (red) k-mer counts with three different methods: Median of Ratios (MR), Trimmed Mean or M-values (TMM) and Upper quartile (UQ). (**c**) ROC plots indicate the detection of known fold-change differences between the mock communities following normalization with synthetic ladder (blue), sample (red), or unnormalized (grey) in simulated (**upper panel**) or experimental (**lower panel**) libraries. A total of 19,768 k-mers from 9 different bacterial species (see Methods) mixed at known fold changes were used to evaluated the normalization performance between communities A & C. In **b**, where data is represented as boxplots, the middle line is the median, the lower and upper hinges correspond to the first and third quartiles, the upper whisker extends from the hinge to the largest value no further than 1.5 x IQR from the hinge (where IQR is the inter-quartile rage) and the lower whisker extends from the hinge to the smallest value at most 1.5 x IQR of the hinge and any points beyond the whiskers are represented individually.

Supplementary Tables

Supplementary Table 1. Stoichiometry for the bacterial DNA in the different mock microbial samples

RefSeq	SPECIES	SAMPLE A	SAMPLE B	SAMPLE C
NZ_CP023689.1	*Streptomyces chartreusis*	15	10	5
NZ_CP023689.1	*Streptomyces nigra*	5	5	5
NZ_CP009124.1	*Streptomyces lividans*	15	10	5
NZ_FOBF00000000.1	*Nonomuraea pusilla*	15	35	30
NZ_MAGP00000000.1	*Micromonospora chalcea*	5	5	5
NZ_LT607754.1	*Micromonospora inositola*	15	10	30
NZ_CP024985.1	*Streptomyces lavendulae*	5	5	5
NZ_JOBA00000000.1	*Streptomyces resistomycificus*	5	5	5
NC_000913.3	*Escherichia coli*	5	5	5

Supplementary Table 2. Stoichiometry for mixtures A, B and C

RefSeq	SCAFFOLD	START	END	MIX.A	MIX.B	MIX.C
NZ_CP023689.1	NZ_JH164838.1	1	100000	8	6	4
NZ_CP023689.1	NZ_JH164860.1	1	100000	4	1	2
NZ_CP023689.1	NZ_JH164868.1	1	100000	4	2	4
NZ_CP023689.1	NZ_JH164837.1	100001	200000	1	1	1
NZ_CP023689.1	NZ_JH164852.1	100001	200000	4	2	4
NZ_CP023689.1	NZ_JH164855.1	200001	300000	4	1	2
NZ_CP023689.1	NZ_JH164841.1	100001	200000	1	1	2
NZ_CP023689.1	NZ_JH164845.1	1	100000	8	6	4
NZ_CP023689.1	NZ_JH164870.1	1	100000	4	6	4
NZ_CP023689.1	NZ_JH164848.1	200001	300000	4	7	4
NZ_CP029043.1	NZ_CP029043.1	6700001	6800000	2	1	4
NZ_CP029043.1	NZ_CP029043.1	1400001	1500000	4	2	4
NZ_CP029043.1	NZ_CP029043.1	5400001	5500000	8	4	4
NZ_CP029043.1	NZ_CP029043.1	4700001	4800000	4	6	4
NZ_CP029043.1	NZ_CP029043.1	1	100000	2	1	2
NZ_CP029043.1	NZ_CP029043.1	4900001	5000000	8	7	4
NZ_CP029043.1	NZ_CP029043.1	3700001	3800000	8	8	8
NZ_CP029043.1	NZ_CP029043.1	1000001	1100000	8	6	4
NZ_CP029043.1	NZ_CP029043.1	6900001	7000000	4	1	2
NZ_CP029043.1	NZ_CP029043.1	5900001	6000000	4	2	4
NZ_CP009124.1	NZ_CP009124.1	3000001	3100000	2	2	2
NZ_CP009124.1	NZ_CP009124.1	4500001	4600000	2	2	4
NZ_CP009124.1	NZ_CP009124.1	2100001	2200000	4	2	4
NZ_CP009124.1	NZ_CP009124.1	1100001	1200000	2	1	2

NZ_CP009124.1	NZ_CP009124.1	6900001	7000000	2	1	4
NZ_CP009124.1	NZ_CP009124.1	3300001	3400000	4	4	8
NZ_CP009124.1	NZ_CP009124.1	700001	800000	8	7	4
NZ_CP009124.1	NZ_CP009124.1	4200001	4300000	4	2	4
NZ_CP009124.1	NZ_CP009124.1	5700001	5800000	4	2	4
NZ_CP009124.1	NZ_CP009124.1	8100001	8200000	4	2	4
NZ_FOBF00000000.1	NZ_FOBF01000018.1	1	100000	4	4	8
NZ_FOBF00000000.1	NZ_FOBF01000002.1	500001	600000	2	1	2
NZ_FOBF00000000.1	NZ_FOBF01000004.1	200001	300000	4	1	4
NZ_FOBF00000000.1	NZ_FOBF01000016.1	1	100000	4	4	8
NZ_FOBF00000000.1	NZ_FOBF01000017.1	100001	200000	4	4	8
NZ_FOBF00000000.1	NZ_FOBF01000015.1	1	100000	4	7	4
NZ_FOBF00000000.1	NZ_FOBF01000022.1	1	100000	4	6	4
NZ_FOBF00000000.1	NZ_FOBF01000006.1	1	100000	4	2	4
NZ_FOBF00000000.1	NZ_FOBF01000012.1	1	100000	2	1	2
NZ_FOBF00000000.1	NZ_FOBF01000027.1	1	100000	4	4	4
NZ_MAGP00000000.1	MAGP01000046.1	1	100000	1	1	1
NZ_MAGP00000000.1	MAGP01000013.1	1	100000	1	1	1
NZ_MAGP00000000.1	MAGP01000075.1	1	100000	1	1	1
NZ_MAGP00000000.1	MAGP01000132.1	1	100000	1	1	1
NZ_MAGP00000000.1	MAGP01000035.1	1	100000	1	1	1
NZ_MAGP00000000.1	MAGP01000121.1	1	100000	1	1	1
NZ_MAGP00000000.1	MAGP01000067.1	1	100000	1	1	1
NZ_MAGP00000000.1	MAGP01000110.1	1	100000	1	1	1
NZ_MAGP00000000.1	MAGP01000001.1	200001	300000	1	1	1
NZ_MAGP00000000.1	MAGP01000002.1	1	100000	1	1	1
NZ_LT607754.1	NZ_LT607754.1	6200001	6300000	2	1	2
NZ_LT607754.1	NZ_LT607754.1	1400001	1500000	2	1	2
NZ_LT607754.1	NZ_LT607754.1	5500001	5600000	2	1	2
NZ_LT607754.1	NZ_LT607754.1	2700001	2800000	1	1	2
NZ_LT607754.1	NZ_LT607754.1	2500001	2600000	2	1	2
NZ_LT607754.1	NZ_LT607754.1	6400001	6500000	4	1	2
NZ_LT607754.1	NZ_LT607754.1	4800001	4900000	4	1	2
NZ_LT607754.1	NZ_LT607754.1	5100001	5200000	4	1	2
NZ_LT607754.1	NZ_LT607754.1	2000001	2100000	2	1	2
NZ_LT607754.1	NZ_LT607754.1	1000001	1100000	2	1	2
NZ_CP024985.1	NZ_JOEW01000051.1	1	100000	2	1	2
NZ_CP024985.1	NZ_JOEW01000014.1	1	100000	1	1	2
NZ_CP024985.1	NZ_JOEW01000023.1	100001	200000	4	1	2
NZ_CP024985.1	NZ_JOEW01000027.1	1	100000	2	1	2
NZ_CP024985.1	NZ_JOEW01000012.1	1	100000	2	1	2
NZ_CP024985.1	NZ_JOEW01000002.1	300001	400000	2	1	2

NZ_CP024985.1	NZ_JOEW01000009.1	100001	200000	2	1	2
NZ_CP024985.1	NZ_JOEW01000011.1	100001	200000	2	1	2
NZ_CP024985.1	NZ_JOEW01000019.1	1	100000	2	1	2
NZ_CP024985.1	NZ_JOEW01000029.1	1	100000	1	1	2
NZ_JOBA00000000.1	NZ_KL575613.1	1	100000	2	1	2
NZ_JOBA00000000.1	NZ_KL575616.1	1	100000	4	1	2
NZ_JOBA00000000.1	NZ_KL575585.1	100001	200000	2	1	2
NZ_JOBA00000000.1	NZ_KL575628.1	1	100000	2	1	2
NZ_JOBA00000000.1	NZ_KL575586.1	100001	200000	2	1	2
NZ_JOBA00000000.1	NZ_KL575589.1	100001	200000	2	1	2
NZ_JOBA00000000.1	NZ_KL575607.1	100001	200000	1	1	2
NZ_JOBA00000000.1	NZ_KL575598.1	1	100000	2	1	2
NZ_JOBA00000000.1	NZ_KL575592.1	1	100000	2	1	2
NZ_JOBA00000000.1	NZ_KL575592.1	100001	200000	1	1	2
NC_000913.3	NC_000913.3	2400001	2500000	8	8	8
NC_000913.3	NC_000913.3	3800001	3900000	8	8	8
NC_000913.3	NC_000913.3	3200001	3300000	8	8	8
NC_000913.3	NC_000913.3	400001	500000	8	8	8
NC_000913.3	NC_000913.3	600001	700000	8	8	8
NC_000913.3	NC_000913.3	3600001	3700000	8	8	8
NC_000913.3	NC_000913.3	4100001	4200000	8	8	8
NC_000913.3	NC_000913.3	2000001	2100000	8	8	8
NC_000913.3	NC_000913.3	200001	300000	8	8	8
NC_000913.3	NC_000913.3	3100001	3200000	8	8	8

Supplementary Notes

1. The supplementary file source_data.1.tab contains the source data to generate Figures 1 and 2. These are the definitions of the columns present in the data table:
    - SAMPLE: Library ID.
    - SEQUENCE: K-mer sequence.
    - LADDER: Synthetic ladder ID.
    - COPY: Copy-number (cn).
    - COUNT: Count observed in the library.
    - TYPE: Sequencing platform.
    - LIBRARY_PREP: Library preparation kit.
2. The supplementary file source_data.2.tab contains the source data to generate Figures 3. These are the definitions of the columns present in the data table:
    - SEQUENCE: K-mer sequence.
    - COUNT: Count observed in the library.
    - SAMPLE: Library ID.
    - CHROM: corresponding human chromosome or Synthetic ladder.
    - POS: position relative to hg38 or the the synthetic ladder.
    - COPY: homozygous or heterozygous for NA12878 and copy-number for Synthetic ladder.
    - ORIGIN: NA12878 or Synthetic ladder.
3. The supplementary file source_data.3.tab contains the source data to generate Figures 4. These are the definitions of the columns present in the data table:
    - SEQUENCE: K-mer sequence.
    - S1: count for sample 1 in the comparison.
    - S2: count for sample 2 in the comparison.
    - CATEGORY: Het x Het, Hom x Het, Het x Hom and Hom x Hom for genomic k-mers and copy-number for ladder k-mers.
    - P.VALUE: p-value for one-sided t-test.
    - P.VALUE.ADJ: adjusted p-value using 0.05 FDR.
    - COMPARISON: son x mother, son x father or father x mother.
    - ORIGIN: K-mer origin (Genomic or Ladder).
    - SAMPLES: Libraries IDs.
4. The supplementary file source_data.4.tab contains the source data to generate Figures 5. These are the definitions of the columns present in the data table:
    - SEQUENCE: K-mer sequence.
    - S1: count for sample 1 in the comparison.
    - S2: count for sample 2 in the comparison.
    - EXPECTED_FOLD: expected fold difference between samples.
    - P.VALUE: p-value for one-sided t-test.
    - P.VALUE.ADJ: adjusted p-value using 0.05 FDR.
    - COMPARISON: son x mother, son x father or father x mother.
    - ORIGIN: K-mer origin (Meta or Ladder).
    - METHOD: Unnormalized, normalized using the ladder or normalized without the ladder.
    - NORMALIZATION: Median ratios, TMM or Upper Quartile.
