# Supplementary material for: A universal and independent synthetic DNA ladder for the quantitative measurement of genomic features
Source: Nat Commun. 2020 Jul 17;11:3609. doi: 10.1038/s41467-020-17445-5 (PMC7367866; doi:10.1038/s41467-020-17445-5)
Supplement: Supplementary file 3 — Reporting Summary [file 41467_2020_17445_MOESM3_ESM.pdf]

nature research

Corresponding author(s): Dr Tim Mercer

Last updated by author(s): Jun 18, 2020

Reporting Summary

Nature Research wishes to improve the reproducibility of the work that we publish. This form provides structure for consistency and transparency in reporting. For further information on Nature Research policies, see Authors & Referees and the Editorial Policy Checklist .

Statistics

For all statistical analyses, confirm that the following items are present in the figure legend, table legend, main text, or Methods section.

n/a	Confirmed	
[ ]	[x]	The exact sample size (*n*) for each experimental group/condition, given as a discrete number and unit of measurement
[ ]	[x]	A statement on whether measurements were taken from distinct samples or whether the same sample was measured repeatedly
[ ]	[x]	The statistical test(s) used AND whether they are one- or two-sided<br>*Only common tests should be described solely by name; describe more complex techniques in the Methods section.*
[x]	[ ]	A description of all covariates tested
[x]	[ ]	A description of any assumptions or corrections, such as tests of normality and adjustment for multiple comparisons
[ ]	[x]	A full description of the statistical parameters including central tendency (e.g. means) or other basic estimates (e.g. regression coefficient) AND variation (e.g. standard deviation) or associated estimates of uncertainty (e.g. confidence intervals)
[ ]	[x]	For null hypothesis testing, the test statistic (e.g. *F*, *t*, *r*) with confidence intervals, effect sizes, degrees of freedom and *P* value noted<br>*Give P values as exact values whenever suitable.*
[x]	[ ]	For Bayesian analysis, information on the choice of priors and Markov chain Monte Carlo settings
[x]	[ ]	For hierarchical and complex designs, identification of the appropriate level for tests and full reporting of outcomes
[ ]	[x]	Estimates of effect sizes (e.g. Cohen's *d*, Pearson's *r*), indicating how they were calculated

Our web collection on statistics for biologists contains articles on many of the points above.

Software and code

Policy information about availability of computer code

Data collection	None.
Data analysis	Custom code used in the analysis can be found at: https://github.com/almreis/Synthetic_Ladder.<br>We also used the following software: wgsim (version 1.9), jellyfish (version 2.2.10), seqtk (version 1.0-r82-dirty),R (v3),ONT Albacore Sequencing Pipeline Software (version 1.2.6), DeSeq2 (version 1.24.0), edgeR (version 3.26.0).

For manuscripts utilizing custom algorithms or software that are central to the research but not yet described in published literature, software must be made available to editors/reviewers. We strongly encourage code deposition in a community repository (e.g. GitHub). See the Nature Research guidelines for submitting code & software for further information.

Data

Policy information about availability of data

All manuscripts must include a data availability statement. This statement should provide the following information, where applicable:

- Accession codes, unique identifiers, or web links for publicly available datasets
- A list of figures that have associated raw data
- A description of any restrictions on data availability

Associated data files for whole genome sequencing and metagenomics analyses can be downloaded from SRA (Accesion numbers: PRJNA625156 and PRJNA625162) and any other synthetic sequences, variant annotations and sequencing libraries can be accessed at http://www.sequinstandards.com.

Field-specific reporting

Please select the one below that is the best fit for your research. If you are not sure, read the appropriate sections before making your selection.

Life sciences study design

All studies must disclose on these points even when the disclosure is negative.

Sample size	To validate the use of Synthetic DNA ladders, as reference standards, it is important to establish reproducibility and commutability in measuring technical variation in sequencing experiments. Therefore, to demonstrate reproducibility, we extensively sequenced Synthetic DNA ladder mixtures under the same experimental conditions. For example, we had 5 independent sequencing experiments of DNA ladders on Illumina HiSeq X Ten and prepared with KAPA HyperPlus kit, 5 independent sequencing experiments of DNA ladders on Illumina HiSeq 2500 and prepared with Nextera XT kit and 4 independent sequencing experiments of DNA ladders on Illumina HiSeq 2500 and prepared with target capture. The sample size in these conditions is sufficient to demonstrate that the variation measured by the DNA ladders is reproducible under the same experimental conditions and specific to different sequencing platforms and library preparation protocols. To demonstrate commutability, we performed 7 WGS experiments of well-characterised genomes accompanied with DNA ladders. Four of those experiments were on the same sample, NA12878, and the other 3, on the Jewish trio (NA24149, NA24143 and NA24385). In all the WGS experiments, we were able to show that the DNA ladder was commutable to the accompanying sample, by comparing its different copy-numbers to known high-confidence variants (heterozygous or homozygous) and showing that they exhibited the same level of variation.
Data exclusions	No data was excluded without description in the Methods section.
Replication	The DNA ladders are a reference to measure technical variation in sequencing experiments, due to the constant copy-number (cn) of its different sequence elements. We sequenced over 20 libraries containing the DNA ladders, and although the variation was different depending on different variables such as sequencing platform, library preparation kit, sequencing error, library depth and library complexity, the fundamental feature of DNA ladders (constant internal ratios) was observed in all of them. Therefore, there are not reported findings that failed to reproduce upon replication.
Randomization	In our experimental design, we were not testing the effect of a treatment on different experimental groups. Our aim was to validate the concept and the use of Synthetic DNA ladders in different experimental conditions, fundamentally demonstrating reproducibility and commutability. Therefore, randomization was not relevant in our study.
Blinding	We did not test the effect of a treatment on different experimental groups. Blinding is fundamental to eliminate experimental biases in that context. However, that is not relevant in our study, especially, since our aim was to be able to measure technical variation in different experimental conditions.

Reporting for specific materials, systems and methods

We require information from authors about some types of materials, experimental systems and methods used in many studies. Here, indicate whether each material, system or method listed is relevant to your study. If you are not sure if a list item applies to your research, read the appropriate section before selecting a response.

Materials & experimental systems

n/a	Involved in the study	
☒	☐	Antibodies
☐	☒	Eukaryotic cell lines
☒	☐	Palaeontology
☒	☐	Animals and other organisms
☒	☐	Human research participants
☒	☐	Clinical data

Methods

n/a	Involved in the study	
☒	☐	ChIP-seq
☒	☐	Flow cytometry
☒	☐	MRI-based neuroimaging

Eukaryotic cell lines

Policy information about cell lines

Cell line source(s)	Cell lines GM12878, GM24385, GM24149 and GM24143 were purchased from Coriell Institute of Medical Research.
Authentication	WGS data from each cell line was checked against known high-confidence genotypes.
Mycoplasma contamination	The mycoplasma testing was performed by Coriell (https://www.coriell.org/0/sections/support/global/qcmyco.aspx?pgid=411) and the result was negative for all the cell lines (https://www.coriell.org/0/sections/support/global/qccells.aspx?pgid=409).
Commonly misidentified lines (See ICLAC register)	*Name any commonly misidentified cell lines used in the study and provide a rationale for their use.*
